# Photoepicutaneous tests: a series of 37 Brazilian patients^⋆^^⋆⋆^

**DOI:** 10.1016/j.abd.2020.03.013

**Published:** 2020-08-16

**Authors:** Maria Antonieta Rios Scherrer, Vanessa Barreto Rocha

**Affiliations:** Dermatology Sector, Hospital das Clínicas, Universidade Federal de Minas Gerais, Belo Horizonte, MG, Brazil

Dear Editor,

Photoepicutaneous tests or photopatch tests (PPT) are indicated for the diagnosis of allergic contact photodermatitis (ACPD), a delayed hypersensitivity reaction that occurs when a photoallergen is applied to the skin that is subsequently exposed to ultraviolet (UV) and/or visible radiation.^1^, ^2^, ^3^ Its incidence is unknown; it is estimated to range from 2% to 10% of patients referred for investigation of photodermatoses.^4^

Patients treated at the Contact Dermatitis Outpatient Clinic, Dermatology Annex, Hospital das Clínicas, UFMG between 2007 and 2019, with diagnostic suspicion of photodermatosis due to eczematous lesions in exposed areas, exacerbated after sun exposure, were selected to undergo PPT for diagnostic purposes after the epicutaneous test with the standard Brazilian panel revealed no clinical relevance. Allergens from FDA-Allergenic, Rio de Janeiro and Chemotechnique Diagnostics, Sweden (Table 1) were used in Finn Chambers® with Scanpor® (Smartpractice, United States) or Allergochambers® (Neoflex, São Paulo) applied in duplicate on the skin of the back. The set of allergens tested was chosen based on the literature and availability in this service.^1^, ^2^, ^3^, ^4^, ^5^ The allergen panel was removed after 48 h, read, and covered with surgical dressing and aluminum foil. As no patient presented a minimal erythematous dose less than 10 J/cm^2^ of UVA, the other set of allergens was irradiated with 10 J, followed by immediate reading and subsequent occlusion. New readings were performed 48 h later, following the International Contact Dermatitis Research Group criteria.^5^ If only the irradiated side showed a positive reaction, the diagnosis of photoallergic contact reaction was made. If both sides had positive reactions, but there was a stronger reaction on the irradiated side, the diagnosis was photodermatitis and allergic contact dermatitis; if the reactions were equal on both sides, the diagnosis was allergic contact dermatitis (ACD).^5^

**Table 1:** Panel of photoepicutaneous tests and number of photoallergic reactions

Hapten	Vehicle	Number of photoallergic reactions
PABA 10%	Vaseline	0
Oxybenzone (benzophenone-3) 10%	Vaseline	3
Butyl methoxybenzoylmethane 10%	Vaseline	1
Sesquiterpene lactone mix 0.1%	Vaseline	1
Musk xylene 1%	Vaseline	0
Balsam of Peru 35%	Vaseline	6
Promethazine 1%	Vaseline	4
Perfume mix 7%	Vaseline	5
Irgasan 1%	Vaseline	0
Potassium bichromate 0.5%	Vaseline	4
Chlorpromazine hydrochloride 0.1%	Vaseline	9
Thiourea 0.1%	Vaseline	1
Chlorhexidine 0.5%	Water	4
Paraphenylenediamine 1%	Vaseline	2
*Compositae* mix 5%	Vaseline	2
Butyl hydroxytoluene (BHT) 2%	Vaseline	3

Among 1712 patients undergoing epicutaneous tests, 37 (2.2%) were selected: 19 men (51.4%) and 18 women (48.6%), aged 30–80 years, 22 (59.4%) of phototypes II and III, seven (19%) of IV and V, and eight (21.6%) of VI. Six patients (16.2%) had a history of atopy, 15 (40.5%) used sunscreens, 31 (84%) had varied occupations, four (11%) were bricklayers, and two (5.4%) were farmers. The time of evolution of the lesions ranged from five months to 20 years and the most affected sites were exposed areas, predominantly the face (33%–89%), upper limbs (25%–67%), and neck (24%–65%). Three patients (8%) had disseminated lesions. Prior patch test indicated Kathon and quaternium-15 reaction in one patient, formaldehyde in one, and potassium bichromate in two; one patient reacted to nickel, thimerosal, and hydroquinone. A total of 74 positive reactions were observed, 54 on the irradiated side and 20 on the non-irradiated side, which led to the diagnosis of ACDP in 23 (62%) patients, and ACD in 12 (32%) (Figure 1, Figure 2). The photoallergic reactions detected were due to chlorpromazine (*n* = 9; 24%), balsam of Peru (*n* = 6; 16%), perfume mix (*n* = 5; 13.5%), promethazine, chlorhexidine, and potassium bichromate (*n* = 4; 11%), oxybenzone and BHT (*n* = 3; 8%), paraphenylenediamine and *Compositae* mix (*n* = 2; 5.4%), sesquiterpene lactone mix, thiourea, and butyl methoxybenzoylmethane (*n* = 1; 2.7%). Six patients (16%) tested negative on both sides.

**Figure 1:**
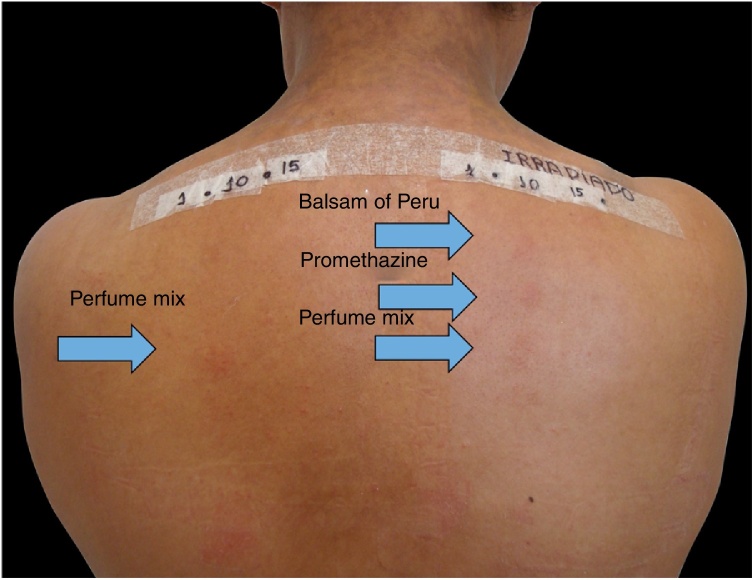
Epicutaneous photo test: DAC to perfume mix and allergic contact photodermatitis (ACPD) to balsam of Peru and promethazine.

**Figure 2:**
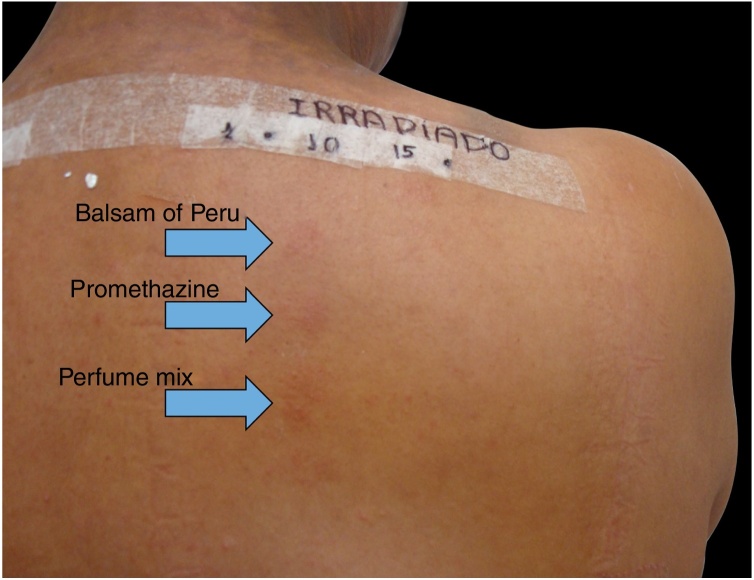
Positive reactions on the irradiated side of the photo epicutaneous test.

Positive reactions to PPT are mainly caused by topical anti-inflammatory drugs such as ketoprofen, widely used in Europe. Studies show co-sensitization with benzophenones, fenofibrate, and perfume mix. Reactions to other anti-inflammatory drugs, such as etofenamate and diclofenac, have been described in the literature. These are the most frequent photoallergens in Europe.^2^, ^4^

In the United States, sunscreens and antibacterials are the most frequent photoallergens, and a progressive reduction in the incidence of fragrances is being observed. In India, *Parthernium* is the most common.^3^ There are no data published in Brazil.

In the present patients, reactions to chlorpromazine were the most frequent, similarly to the findings of a Chinese series.^1^ It is an antipsychotic derived from phenothiazine whose analogs, such as dihydrochlorothiazide and promethazine, are widely used as diuretics and antipruritics in Brazil.^1^ Promethazine was the fourth most common photoallergen, tied for fourth place with chlorhexidine and potassium bichromate, but only one patient showed a co-reaction between chlorpromazine and promethazine. The second in frequency was balsam of Peru, followed by perfume mix. In the Chinese research, potassium bichromate was also the fourth most frequently identified photoallergen, showing one more similarity with the present study, in addition to chlorpromazine.^1^

The pattern of photopositivity varies according to the area and population studied. This is a preliminary investigation, which reflected the population's habits, such as the use of oral and topical promethazine as an antipruritic, and professional or domestic exposure to cement.

The authors suggest that a standard PPT panel should be defined for Brazil. This set of allergens used is a preliminary suggestion to establish a Brazilian panel for photoepicutaneous tests, since it is an effective and important tool in the diagnosis of photosensitive dermatitis.

## Financial support

None declared.

## Authors’ contributions

Maria Antonieta Rios Scherrer: Statistical analysis; approval of the final version of the manuscript; conception and planning of the study; elaboration and writing of the manuscript; obtaining, analyzing, and interpreting the data; intellectual participation in propaedeutic and/or therapeutic conduct of studied cases; critical review of the literature; critical review of the manuscript.

Vanessa Barreto Rocha: Approval of the final version of the manuscript; obtaining, analyzing, and interpreting the data; intellectual participation in propaedeutic and/or therapeutic conduct of studied cases; critical review of the literature; critical review of the manuscript.

## Conflicts of interest

None declared.
